# Nickel Coarsening and Mass Transfer Performance Prediction in Direct Internal Reforming Solid Oxide Fuel Cells

**DOI:** 10.3390/nano16100633

**Published:** 2026-05-20

**Authors:** Xiaoxing Yang, Guogang Yang, Hao Wang, Han Sun, Zhuangzhuang Xu, Shengzheng Ji

**Affiliations:** Marine Engineering College, Dalian Maritime University, Dalian 116000, China; yangxiaoxing@dlmu.edu.cn (X.Y.); whdlmu@dlmu.edu.cn (H.W.); sunhandlmu@dlmu.edu.cn (H.S.); zzxu99@dlmu.edu.cn (Z.X.); loki971125@dlmu.edu.cn (S.J.)

**Keywords:** Ni coarsening, mass transfer, phase-field modeling, lattice Boltzmann modeling, solid oxide fuel cell anode

## Abstract

Ni coarsening is a primary degradation mechanism in Ni-based anodes, significantly contributing to performance decline and diminished lifespan of methane steam reforming solid oxide fuel cells (SOFCs) during long-term operation. In this study, a novel algorithm is introduced to reconstruct two-dimensional Ni-YSZ anode microstructures, complemented by the development of a multi-physics model that integrates phase-field modeling (PFM) with the Lattice Boltzmann Method (LBM). This coupled PFM-LBM framework is employed to investigate the effects of Ni agglomeration on microstructural evolution and methane-steam mass transport under diverse conditions. The results demonstrate that the initial Ni particle diameter exerts a significant influence on Ni agglomeration dynamics. Furthermore, the mass transport analysis reveals that the necking structures formed during Ni coarsening pose a substantial impediment to mass transfer efficiency. Finally, optimized structural parameters for Ni-YSZ are proposed to enhance anode performance in Ni-based electrodes.

## 1. Introduction

As the target of carbon reduction is deeply ingrained in humanity’s mind, many countries have proposed policies to reduce carbon emissions, such as China’s dual carbon goals of “peak carbon” by 2030 and “carbon neutral” by 2060, announced in September 2020 [1]. This means that clean and efficient energy conversion technologies are necessary to meet these requirements. Solid Oxide Fuel Cells (SOFCs) are regarded as an alternative device due to their high efficiency of 60–80%, fuel flexibility, and superior performance [2].

Despite these advantages, several degradation phenomena were detected in the SOFC electrodes [3]. Among the degradation mechanisms, Ni coarsening in the anode has been identified as a major contributor to performance degradation, primarily due to its adverse effects on active site availability and mass transport [4,5,6]. There have been several investigations into the nickel particle growth mechanism and the influence of Ni coarsening on SOFC performance. S. Koch et al. [7] studied the degradation of SOFCs at 750 °C and 850 °C in hydrogen with 5% or 50% water, with current densities ranging from 0.25 A cm^−2^ to 1 A cm^−2^ for 300 h. The investigation revealed that Ni redistribution results in poorer performance, as some of the electrochemically active sites are lost, and that a high anode gas water content especially accelerates this process. In addition, D. Hari Prasad et al. [8] investigated the Ni-based anode for steam reforming of methane in SOFCs; they found that sintering of nickel nanoparticles decreased reforming activity at higher steam-to-carbon ratios. Meanwhile, similar phenomena were observed in other Ni-based SOFCs [9,10,11,12,13,14,15,16]. However, the morphological evolution of Ni coarsening is difficult to capture experimentally. The computational model is ideally suited for elucidating the nickel particle growth mechanism through controlling microstructural evolution processes in SOFC anodes. According to current research, there are three modes for nickel aggregation simulation: two-particle systems [17], phase-field models (PFM) [18,19], and multi-scale frameworks [20]. The phase-field approach is one of the most powerful tools for modeling complex microstructure evolution, particularly the growth of nickel particles in the anode of SOFCs [21,22,23]. Trini, M et al. [24] investigated the Ni coarsening in complex 3D structures of SOC fuel electrodes. The Ni mean radius, triple-phase boundaries, and interface shape distribution were simulated using phase-field modeling. Zhu et al. [25] developed a transient multi-physical field model to investigate the influence of Ni coarsening on SOFC performance. The results showed that high operating temperature significantly accelerates Ni particle growth. Accurate microstructure reconstruction and reliable numerical modeling results not only can correctly elucidate the morphological evolution of a given Ni-based anode cermet structure but also guide the design of the microstructure to improve the lifetime of fuel electrodes. There are two approaches to reconstructing the microstructure of SOFC anodes: experiments [16,24,26,27,28] and algorithms [29,30,31,32]. However, algorithmic reconstruction is more cost-effective and more flexible for controlling porosity, curvature, and pore size.

Although numerous studies have investigated Ni-based SOFC anodes using phase-field modeling, the impact of Ni coarsening on mass transport has received relatively little attention. The Lattice Boltzmann Method (LBM) has been recognized as an accurate and efficient approach for simulating mass transport in anisotropic microstructures [33,34,35]. Several investigations have employed LBM to simulate mass transport mechanisms in SOFC anodes [36,37,38,39]. However, integrated approaches that couple PFM with LBM to explore the impact of Ni coarsening remain scarce. Such combined modeling efforts are essential for elucidating the underlying mechanisms by which Ni particle coarsening influences mass transport behavior.

In this study, a multi-physics model for the Ni-based anode of SOFCs is developed by coupling Ni coarsening and mass transport. The porous Ni-YSZ anode is reconstructed using a stochastic cluster-based algorithm, based on porous parameters obtained from experiments [40]. Subsequently, the PFM is employed to simulate the nickel particle coarsening process and generate the corresponding microstructure, which is then used to construct the physical model of LBM for simulating mass transfer in the porous anode of the SOFC. Therefore, the proposed framework enables a quantitative investigation of SOFC performance degradation under varying anode microstructures. This integrated approach facilitates the efficient optimization of key Ni–YSZ microstructural parameters. Moreover, it provides a practical tool for rapid screening of optimal structures for high-performance SOFC design.

## 2. Model Development

### 2.1. Porous Anode Mode Development

**Figure 1:**
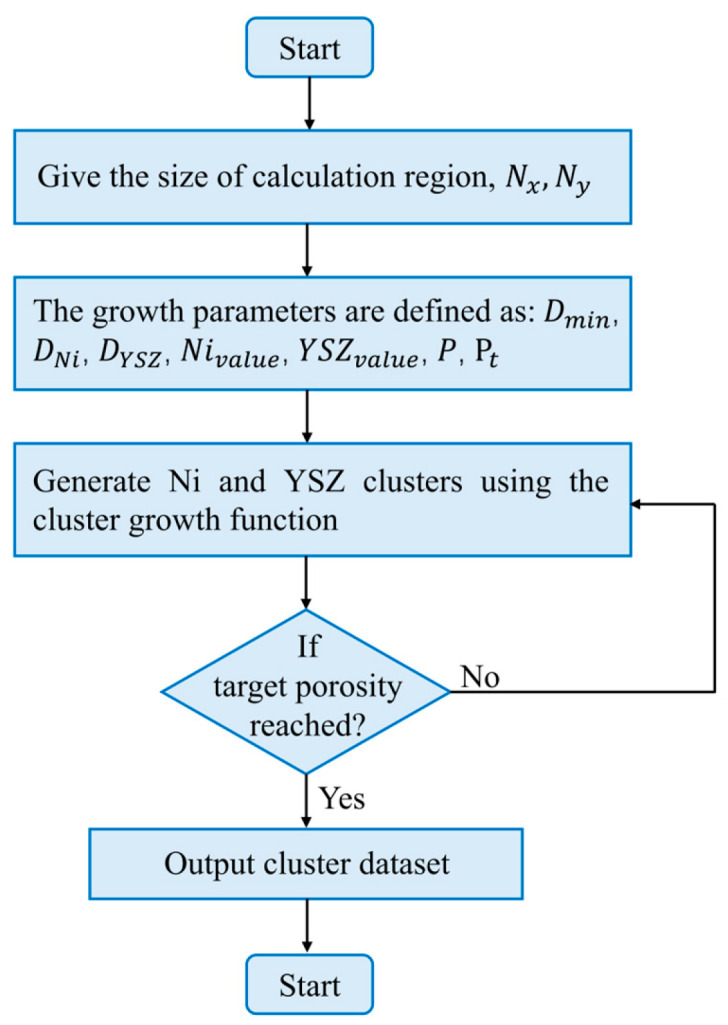
Flowchart of the stochastic cluster-based algorithm.

A stochastic cluster-based algorithm (SCBA) was developed to generate two-dimensional microstructures of Ni–YSZ anodes, as illustrated in Figure 1. The particle diameters of Ni and YSZ used in this algorithm are referenced from statistical measurements of the actual Ni–YSZ anode, with mean sizes of 1 μm (accounting for 46%) and 0.6 μm (accounting for 36%), respectively, within a cubic domain of 6 μm × 6 μm × 6 μm [40]. Although pore networks in SOFC anodes are typically well connected in three-dimensional space, disconnections may appear in two-dimensional slices. A non-adjacent clustering strategy was adopted to ensure adequate spacing between particles, thus facilitating pore formation and interconnectivity. This algorithm enables the generation of microstructures with controllable material distributions and specified porosity. Specifically, the spatial distribution of nickel and yttria-stabilized zirconia (YSZ) clusters can be tailored in the 2D domain, allowing critical microstructural characteristics of the SOFC anode, such as porosity and Ni and YSZ particle diameters, to be accurately reconstructed and systematically controlled. The governing equation for the anode porosity is given as follows.

(1) $$\phi_{A}=1-\frac{A_{0}}{A_{\mathrm{total}}}$$

Here, $A_{0}$ represents the area occupied by Ni and YSZ, and $A_{total}$ denotes the total area of the computational domain.

### 2.2. Phase Field Model for Ni Coarsening

A phase-field model with three primary phases (Ni, YSZ, and pores) was developed to describe the coarsening of Ni-YSZ in the SOFC anode. The YSZ phase is treated as stationary due to its extremely slow diffusion rate [25]. A set of variables (conserved and non-conserved order parameters) is used to represent the concentration field and the phase order field, respectively. In the Ni–YSZ anode, conserved variables represent the volume fractions of each constituent and satisfy the constraint $c_{Ni}+c_{YSZ}+c_{pore}=1$ Non-conserved variables, denoted as order parameters, are introduced to describe the Ni phase (φ=1) and the pore phase (φ=0) The total free energy consists of the local free energy density and interfacial energy contributions and can be expressed as follows [23].

(2) $$F(c,\varphi )=\int \left[f_{0}(c,\varphi )+\frac{\varepsilon_{c}}{2}{\left|\nabla c\right|}^{2}+\frac{\varepsilon_{\varphi }}{2}{\left|\nabla \varphi \right|}^{2}\right]ds.$$

Here, $f_{0}$ denotes the local free energy density, and ε represents the gradient energy coefficient used to control the interfacial thickness. The local free energy is described employing a double-well potential function as follows.

(3) $$f_{0}(c,\varphi )=\sum_{i}f_{1}(c)+\sum_{i}f_{2}(c,\varphi ).$$

where

(4) $$\left\{\begin{array}{l} f_{1}(c)=-\frac{w}{4}c^{2}{\left(1-c\right)}^{2}\\ f_{2}(c,\varphi )=-\frac{1}{2}c^{2}\varphi^{2}+\frac{1}{4}\varphi^{2} \end{array}\right..$$

Here, w refer to the well depth.

The reduction in total free energy drives the morphological evolution of the phases. The temporal evolution of the phase volume fractions and grain orientations is governed by the Cahn–Hilliard and the Allen–Cahn equations, respectively.

The coarsening behavior of Ni is described by the Cahn–Hilliard equation, which describes the temporal evolution of the Ni concentration field, as follows.

(5) $$\frac{\partial c}{\partial t}=\nabla \cdot \left(M_{c}\nabla \mu \right).$$

(6) $$\mu =\frac{\partial f(c,\varphi )}{\partial c}-\kappa_{c}\nabla^{2}c.$$

where $M_{c}$ is the concentration-dependent diffusion coefficient describing the mobility of Ni within the YSZ matrix. μ represents the chemical potential, defined as the variational derivative of the free energy with respect to the Ni concentration. $\kappa_{c}$ is the constant controlling the intensity of Ni diffusion.

The Allen–Cahn equation is employed to capture the interfacial evolution during the Ni coarsening process by describing the temporal variation of the phase-field variable φ.

(7) $$\frac{\partial \varphi }{\partial t}=-M_{\varphi }\frac{\delta F}{\delta \varphi }.$$

(8) $$\frac{\delta F}{\delta \varphi }=\frac{\partial f(c,\varphi )}{\partial \varphi }-\kappa_{\varphi }\nabla^{2}\varphi .$$

where $M_{\varphi }$ represents the interfacial mobility, controlling the rate of interface migration between Ni and YSZ. $\frac{\delta F}{\delta \varphi }$ Corresponds to the functional derivative of the total free energy with respect to the phase-field variable. The evolution of the interface is driven by the tendency to minimize the total free energy.

### 2.3. Lattice Boltzmann Model for Mass Transfer

The Boltzmann equation governing multi-species reaction-diffusion processes in the non-continuum regime is formulated as follows [33].

(9) $$f^{i}(x+e^{i}\Delta t,t+\Delta t)=f^{i}(x,t)+\Omega^{ii}+\sum_{\begin{array}{l} j=1\\ j\neq i \end{array}}^{n}\Omega^{ij}++\Omega_{\alpha }^{ik}.$$

where $f^{i}(x,t)$ denotes the density distribution function of the ith species at position x and time t. $e^{i}$ represents the velocity of the ith species, and Δt is the time interval. Superscript represents $CH_{4}$ or $H_{2}O$. The term $\Omega^{ii}$ represents the self-collision among particles of the same species, while the cross-collision term $\Omega^{ij}$ describes the interactions between the ith species and other species.

The following equation is derived through the discretization of Equation (5).

(10) $$f_{\alpha }^{i}(x+e_{\alpha }^{i}\Delta t,t+\Delta t)=f_{\alpha }^{i}(x,t)+\Omega_{\alpha }^{ii}+\sum_{\begin{array}{l} j=1\\ j\neq i \end{array}}^{n}\Omega_{\alpha }^{ij}++\Omega_{\alpha }^{ik}.$$

Here, α represents the direction of the discrete velocity. The D2Q9 discrete velocity model is employed for two-dimensional calculations. The equilibrium distribution function $f_{i}^{1(0)}$, the self-collision term $\Omega_{\alpha }^{ii}$, and the cross-collision term $\Omega_{\alpha }^{ik}$ are defined by the following equations, respectively.

(11) $$\Omega_{\alpha }^{ii}=-\frac{1}{\tau_{i}}\left[f_{\alpha }^{i}-f_{\alpha }^{i(0)}\right].$$

(12) $$\Omega_{\alpha }^{ij}=-\frac{1}{\tau_{ij}}\frac{\rho^{j}}{\rho }\frac{f_{\alpha }^{i(eq)}}{c_{s,i}^{2}}\left(e_{\alpha }^{i}-u\right)\cdot \left(u^{i}-u^{j}\right).$$

(13) $$\Omega_{\alpha }^{ik}=-\frac{1}{\tau_{ik}}\frac{\rho^{k}}{\rho }\frac{f_{\alpha }^{i(eq)}}{c_{s,i}^{2}}\left(e_{\alpha }^{i}-u\right)\cdot \left(u^{i}-u^{k}\right).$$

(14) $$f_{\alpha }^{i(0)}=\left[1+\frac{1}{c_{s,i}^{2}}\left(e_{\alpha }^{i}-u\right)\cdot \left(u^{i}-u\right)\right]f_{\alpha }^{i(eq)}.$$

(15) $$f_{\alpha }^{i(eq)}=\rho^{i}\omega_{\alpha }\left[1+\frac{e_{\alpha }^{i}\cdot u}{c_{s,i}^{2}}+\frac{{\left(e_{\alpha }^{i}\cdot u\right)}^{2}}{2c_{s,i}^{4}}-\frac{u\cdot u}{2c_{s,i}^{2}}\right].$$

(16) $$c_{s,1}=\frac{1}{\sqrt{3}},c_{s,i}=\sqrt{\frac{M_{1}}{M_{i}}}c_{s,1}.$$

Here, $\tau_{i}$ denotes the relaxation time associated with self-collisions, which governs the kinematic viscosity. $\tau_{ij}$ represents the cross-collision relaxation time that determines the species diffusivity, and $C_{s,i}$ is the lattice sound speed for species i. For the D2Q9 model, the parameters $e_{\alpha }^{i}$ and $\omega_{i}$ are given by the following expressions.

(17) $$e_{\alpha }^{i}=c_{i}\left[\begin{array}{ccccccccc} 0 & 1 & 0 & -1 & 0 & 1 & -1 & -1 & 1\\ 0 & 0 & 1 & 0 & -1 & 1 & 1 & -1 & -1 \end{array}\right].$$

(18) $$c_{1}=\frac{\Delta x}{\Delta t},c_{i}=\sqrt{\frac{M_{1}}{M_{i}}}c^{1}.$$

(19) $$\omega_{i}=\left\{\begin{array}{cc} \frac{4}{9}, & i=0\\ \frac{1}{9}, & i=1-4\\ \frac{1}{36}, & \alpha =5-8 \end{array}\right..$$

The molar fraction and velocity vector of each species can be evaluated based on the Chapman–Enskog expansion.

(20) $$\rho^{i}=\sum_{\alpha }f_{\alpha }^{i}=\sum_{\alpha }f_{\alpha }^{i(0)}.$$

(21) $$\rho^{i}u^{i}=\sum_{\alpha }f_{\alpha }^{i}e_{\alpha }^{i}=\sum_{\alpha }f_{\alpha }^{i(0)}e_{\alpha }^{i}.$$

The governing equations for calculating the mixture mass density ρ and bulk velocity u are given as follows.

(22) $$\rho =\sum_{i=1}^{N}\rho^{i}.$$

(23) $$\rho u=\sum_{i=1}^{N}\rho^{i}u^{i}.$$

Equation (10) can be divided into two sequential steps: the collision step and the streaming step. Based on the discretization approach proposed by Xu [33], using an explicit first-order Euler scheme and a second-order Lax–Wendroff scheme on the same computational grid, the resulting formulations are given as follows.

Collision:

(24) $$f_{\alpha }^{i+}\left(x,t\right)=f_{\alpha }^{i}\left(x,t\right)+\Omega_{\alpha }^{ii}+\sum_{\begin{array}{l} j=1\\ j\neq i \end{array}}^{n}\Omega_{\alpha }^{ij}+\Omega_{\alpha }^{ik}.$$

Streaming:

(25) $$f_{\alpha }^{i}\left(x+e_{\alpha }^{i}\Delta t,t+\Delta t\right)=f_{\alpha }^{i+}\left(x,t\right)-\Delta t\left(e_{\alpha }^{i}\cdot \nabla \right)f_{\alpha }^{i+}\left(x,t\right)+\frac{1}{2}\Delta t^{2}\left(e_{\alpha }^{i}\cdot \nabla \right)f_{\alpha }^{i+}\left(x,t\right)+o\left(\Delta t^{3}\right)$$

where the superscript + denotes the post-collision variable.

The binary diffusion coefficient $D_{ij}$ and Knudsen diffusion coefficient $D_{ik}$ are calculated based on the formulations provided in Ref. [41].

(26) $$D_{ij}=\frac{p\rho }{n^{2}M_{i}M_{j}}\left(\tau_{ij}-\frac{1}{2}\right)\Delta t.$$

(27) $$D_{ik}=\frac{p\rho }{n^{2}M_{i}M_{k}}\left(\tau_{ik}-\frac{1}{2}\right)\Delta t.$$

Here, the total amount of substance n in the mixture can be determined from $\rho^{i}/X_{i}=nM_{i}$, and the corresponding pressure relation p is given by the following:

(28) $$p=\sum_{i=1}^{N}\rho^{i}c_{s,i}^{2}$$

### 2.4. Model Verification

A comprehensive validation process was conducted to ensure the reliability of the phase-field model (PFM) for predicting Ni coarsening in the Ni-YSZ anode, focusing on numerical convergence. The LBM model for mass transport has been verified in our previous work [38].

Numerical stability and independence were first assessed to confirm the model’s internal consistency. Grid independence was tested by varying the grid resolution from 100 × 100 to 400 × 400, with the domain size fixed at 6 μm × 6 μm. The average Ni particle diameter after 5000 simulation steps converged within 2% error for resolutions above 200 × 200, validating the chosen grid (200 × 200) as sufficient for accurate simulations without high computational cost. Time step sensitivity was evaluated by reducing dt from 0.00001 s to 0.000001 s. The free energy functional F decreased monotonically, confirming numerical stability and adherence to thermodynamic principles (F decreasing by >95% to equilibrium). Parameter sensitivity analysis was performed on key variables: increasing k from 0.01 to 0.05 accelerated coarsening by 15–20%, consistent with expected surface-energy effects. This validation establishes the robustness of PFM.

## 3. Results and Discussion

### 3.1. Evolution of Ni Coarsening

**Figure 2:**
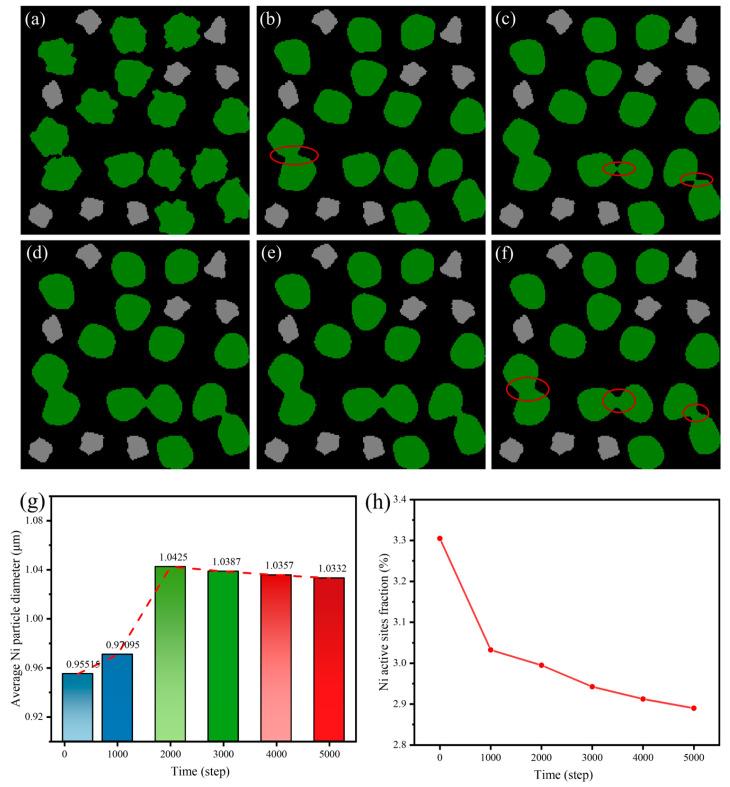
The Ni coarsening evolution at (**a**–**f**) step = 0, 1000, 2000, 3000, 4000, and 5000; (**g**) the nickel particle diameters evolution, and (**h**) the account of Ni active sites in different steps.

The agglomeration behavior of Ni particles significantly influences the electrochemical activity of Ni–YSZ composite anodes. In this section, we focus on the morphological evolution of Ni coarsening at different simulation stages. Figure 2 illustrates the evolution of Ni morphology from 0 to 5000 simulation steps, along with an analysis of the Ni particle diameters and active sites. In the initial state (Figure 2a), Ni particles (green) are distributed as small, dispersed clusters within the YSZ and pore matrix. The particle size is relatively small and uniformly distributed, and the Ni surface appears rough, indicating a large surface area and thus a high density of active sites, which is beneficial for electrochemical reactions. With the progression of the simulation, the coarsening of Ni particles becomes evident, as shown in Figure 2b, where the red-circled region indicates the initial formation of larger agglomerates accompanied by the development of distinct necking structures. Figure 2c captures the second stage of Ni evolution, where additional necking is observed, indicating ongoing coarsening. The early-stage evolution shown in Figure 2a–c occurs rapidly, suggesting that Ni atoms tend to migrate toward lower-energy states, forming larger clusters. As time advances, Figure 2d–f demonstrate the continued agglomeration of Ni particles, albeit at a slower rate. This deceleration is attributed to the Allen–Cahn equation stabilizing phase boundaries during later stages, thereby suppressing the excessive growth of agglomerates. A clear understanding of the Ni coarsening mechanism is essential for the rational design of SOFC anodes, as excessive Ni agglomeration reduces the effective area of the triple-phase boundary (TPB), thereby diminishing electrochemical reaction efficiency.

The average equivalent diameter of Ni particles is calculated and analyzed to quantitatively assess the agglomeration behavior, as shown in Figure 2g. The mean Ni cluster diameter increases from approximately 0.956 μm to around 1.042 μm as the simulation progresses and then reaches a stable value. This trend is consistent with experimental observations of SOFC anodes, where Ni particles grow from submicron to micron scales during prolonged operation, primarily due to surface diffusion at elevated temperatures [10]. The maximum diameter of 1.042 μm indicates a transition to a more heterogeneous anode morphology, which may lead to pore blockage, reduce gas diffusion efficiency, and accelerate anode degradation.

Active sites are critical regions for hydrogen oxidation reactions and gas diffusion. The proportion of active sites is statistically analyzed in Figure 2h. The percentage of active sites decreases linearly from an initial value of 3.305% to a final value of 2.89%, representing a reduction of approximately 0.415%. During the first 2000 simulation steps, the proportion drops rapidly from 3.305% to approximately 2.995%. The decline becomes more gradual and reaches a stable range between 2.89% and 2.9125% from 3000 to 5000 simulation steps. This reduction can be attributed to Ni particle agglomeration driven by surface diffusion and the Ostwald ripening mechanism at elevated operating temperatures, in which smaller particles dissolve and redeposit onto larger Ni clusters, reducing the surface-to-volume ratio. These results indicate that Ni coarsening significantly reduces the number of active sites in the SOFC anode, thereby severely degrading its electrochemical performance.

### 3.2. Effect of Ni Particle Diameter on Coarsening

The effect of Ni particle diameter on coarsening behavior in Ni–YSZ anodes is investigated in this section. Particle size is a key variable that influences coarsening kinetics, agglomerate morphology, and pore connectivity, all of which are closely associated with gas diffusion pathways and triple-phase boundary density. Numerical simulations are conducted for initial Ni particle diameters ranging from 1.20 μm to 0.70 μm. The morphological evolution following coarsening is quantitatively characterized to assess its structural impact and potential implications for anode performance.

**Figure 3:**
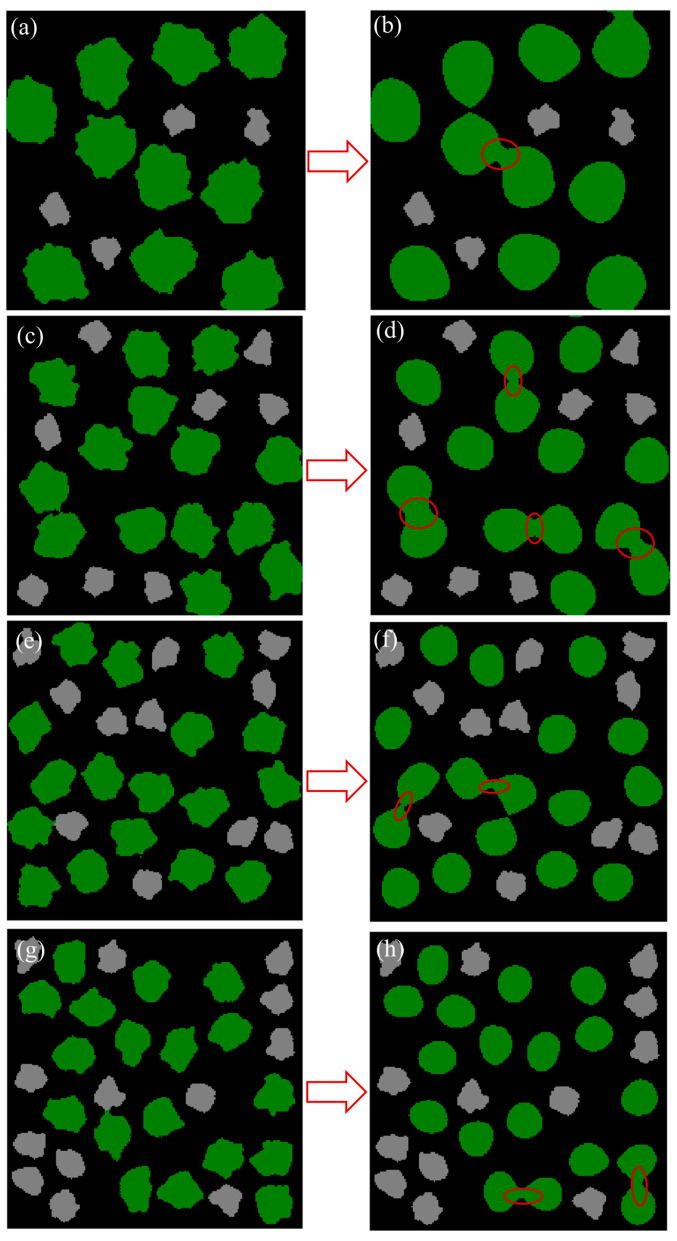
The initial microstructure and the microstructure after 10,000 steps for Ni particle diameters (**a**) 1.20 μm (initial), (**b**) 1.20 μm (after), (**c**) 0.96 μm (initial), (**d**) 0.96 μm (after), (**e**) 0.79 μm (initial), (**f**) 0.79 μm (after), (**g**) 0.70 μm (initial), (**h**) 0.70 μm (after). The green and gray phases are Ni and YSZ, respectively.

**Figure 4:**
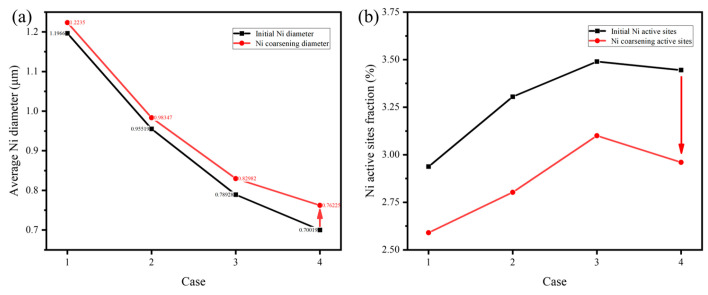
(**a**) The evolution of the Ni average diameter, and (**b**) the fraction of Ni active sites at different initial diameters.

The reconstruction of initial electrodes with different Ni particle diameters is based on the SCBA, where the YSZ particle size and porosity are fixed at 0.6 μm and 60%, respectively. Table 1 summarizes the correspondence between Ni particle diameter and domain size. A 2D pixel grid (200 × 200) is mapped onto a 3D physical domain of 6 μm × 6 μm × 6 μm, and the relative Ni particle size is defined accordingly. The initial and final morphologies for each Ni diameter case after 10,000 simulation steps are compared in Figure 3. The coarsening process for an initial average Ni diameter of 1.20 μm is shown in Figure 3a. The left result illustrates the initial state, where Ni particles are relatively large and sparsely distributed, with clear separation between YSZ and pores. Figure 3b shows the final state, where most Ni particles remain isolated with minimal coalescence. This suggests that a larger initial diameter results in weaker surface-energy-driven diffusion and reduced mobility, leading to slower coarsening. The cluster size increases slightly from 1.20 μm to approximately 1.22 μm, as shown in Figure 4a. Pore connectivity remains high, although the initial TPB density is relatively low. Figure 3c,d correspond to an initial average Ni diameter of 0.96 μm. The initial state shows uniformly distributed particles of intermediate size. In the final state, the red circles highlight moderate coalescence, with the formation of connected clusters and noticeable necking. This indicates a moderate degree of coarsening, with improved cluster connectivity. The average diameter increases to approximately 0.98 μm, accompanied by a slight reduction in the proportion of active sites. Figure 3e,f present the case with an initial average Ni diameter of 0.79 μm. The particles are densely packed in the initial state. The final state exhibits coarsening, with multiple particles merging into large clusters as highlighted by red circles. The average particle size increases substantially to 0.83 μm. Figure 3g,h show the case with an initial diameter of 0.70 μm, where the particles are initially highly dispersed. The final morphology reveals pronounced neck formation and significant agglomeration. This behavior reflects accelerated Ostwald ripening driven by high surface energy at small diameters. In contrast, larger diameters contribute to interface stability and reduced coarsening intensity. Overall, the coarsening tendency increases progressively as the initial Ni particle size decreases.

The evolution of the fraction of active sites as a function of simulation steps for different initial Ni particle diameters is illustrated in Figure 4b. Smaller Ni particle diameters correspond to higher initial TPB densities due to their larger surface-to-volume ratios. However, the active site fraction declines sharply due to Ni particle coarsening. The 0.96 μm and 0.79 μm cases exhibit intermediate behavior, with moderate reductions in the active site fraction, reflecting their balanced coarsening dynamics. The active site fraction decreases by approximately 14.1% after 10,000 steps for the 0.70 μm case, indicating significant structural degradation. These results reveal a distinct size-dependent degradation behavior. Smaller Ni particles exhibit higher initial reactivity due to their larger surface-to-volume ratio, but they are more susceptible to coarsening-induced performance deterioration.

### 3.3. Effect of Ni Coarsening on Gas-Phase Mass Transfer

**Figure 5:**
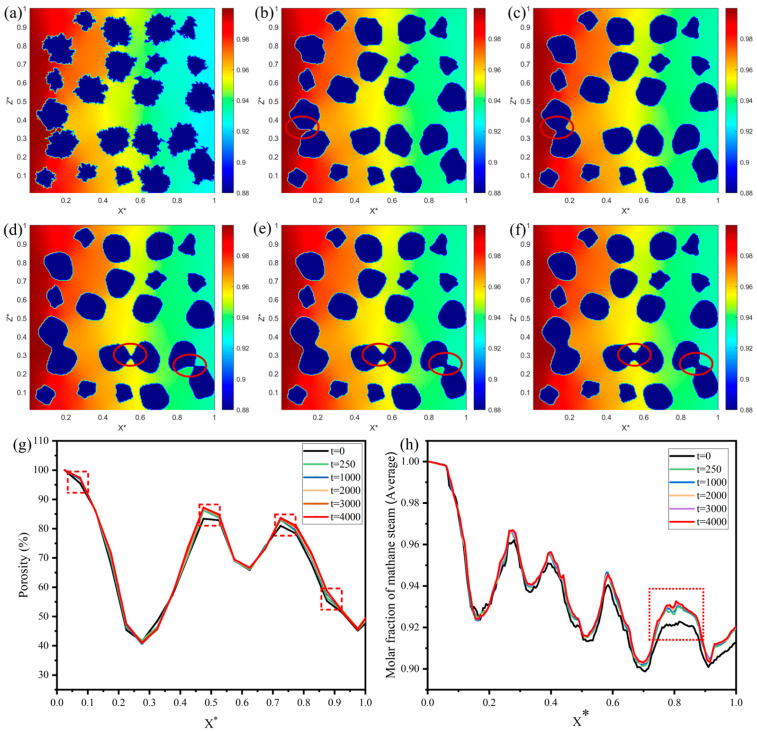
Mole fraction distribution of methane-steam under varying degrees of Ni coarsening: (**a**) at t = 0 steps, (**b**) at t = 250 steps, (**c**) at t = 1000 steps, (**d**) at t = 2000 steps, (**e**) at t = 3000 steps, (**f**) at t = 4000 steps; (**g**) distribution of local porosity, and (**h**) average methane-steam concentration distribution along the X-direction.

Nickel coarsening significantly influences internal gas-phase transport in SOFC anodes, primarily due to associated microstructural degradation. Ni aggregation significantly alters pore connectivity and triple-phase boundary density while directly modulating the diffusion efficiency of fuel gases, such as methane and steam, thereby influencing the electrochemical reaction rate and the overall performance of the anode. To quantitatively investigate the effect of Ni aggregation on gas transport, this section employs coarse-grained structures generated from phase-field simulations to model methane and steam transport under varying degrees of aggregation. The simulation assumes constant boundary conditions with a methane-to-steam molar ratio of 9:1 at the inlet, and the concentration distribution is computed accordingly. Figure 5 presents the gas transport results after 5000 LBM steps at different Ni aggregation levels, along with associated porosity and concentration variations, providing insights into the dynamic impact of Ni aggregation on internal transport within the anode. Figure 5a–f illustrate the evolution of the methane-steam concentration field, with a color gradient ranging from red (high concentration) to blue (low concentration), reflecting the characteristics of mass transport. At the inlet, concentrations are higher, while they diminish toward the outlet. In Figure 5a (t = 0 step, representing the initial Ni particle morphology), the Ni particles are dispersed, and the concentration field is uniformly distributed. High-concentration regions (red) predominantly extend along interconnected pore channels, indicating a high diffusion efficiency. This suggests that the unaggregated structure provides ample connected porosity, facilitating efficient gas diffusion. Figure 5b (t = 1000 steps) reveals that following early-stage aggregation, the concentration field begins to exhibit localized gradient changes. The red-circled region highlights pore constriction due to Ni particle merging, with the high-concentration zone slightly compressed. However, the overall transport pathway remains unobstructed, indicating that mild aggregation imposes only a preliminary hindrance to diffusion. Figure 5c–f demonstrate an accelerated Ni aggregation process. The red-circled regions highlight the formation of necking structures that partially obstruct channels, indicating that Ni coarsening substantially reduces gas permeability at the local scale. This progressive blockage intensifies with increasing simulation steps, underscoring the detrimental effect of aggregation on pore connectivity and mass transport efficiency.

The quantitative analysis of local porosity along the transport direction is conducted to investigate the influence of porosity on methane-steam mass transport, as depicted in Figure 5g. The results reveal that as aggregation progresses, the increase in local porosity elevates the average porosity from an initial 60% to a final 62.36%. This phenomenon arises from the contraction of Ni particles during agglomeration, which releases localized space and consequently augments the overall pore volume. However, this process also introduces localized channel blockages. These results suggest that Ni coarsening partially enhances the pore network configuration. However, this process simultaneously amplifies the propensity for localized blockages, highlighting the imperative for advanced anode design approaches to mitigate transport limitations and enhance overall performance.

The average methane-steam concentration distribution across varying degrees of Ni aggregation, with the curve transitioning from high to low values, is shown in Figure 5h. The overall transport efficiency exhibits a modest enhancement during the later stages of aggregation. This suggests that Ni agglomeration, by forming larger clusters, optimizes macroscopic gas channels, thereby increasing the gas flow rate within the anode. However, the red-circled necking regions indicate complete localized blockage, impeding mass transport. In summary, while Ni aggregation enhances overall transport efficiency, the presence of localized obstructions and the concomitant reduction in active sites at the triple-phase boundary exert a deleterious impact on the performance of the SOFC anode.

**Figure 6:**
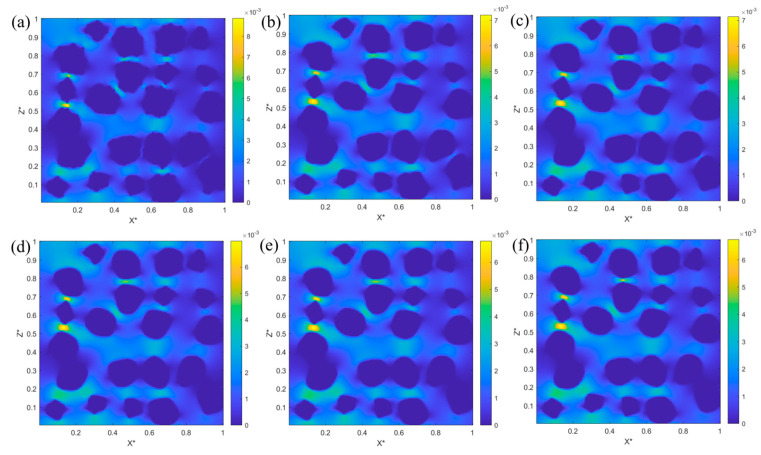
The velocity distribution of methane-steam at different degrees of Ni coarsening: (**a**) t = 0 steps, (**b**) t = 250 steps, (**c**) t = 1000 steps, (**d**) t = 2000 steps, (**e**) t = 3000 steps, (**f**) t = 4000 steps.

The velocity distribution within the anode along the flow direction (X-direction) under varying degrees of Ni coarsening is depicted in Figure 6a–f. Deep blue regions represent the solid Ni and YSZ particles, where the velocity is negligible due to no-slip boundary conditions. The velocity of methane-steam increases significantly within narrow pore channels. However, the flow velocity is substantially diminished in regions characterized by Ni agglomeration, indicating complete obstruction of methane-steam transport. This localized blockage profoundly affects both catalytic performance and mass transport efficiency, ultimately contributing to a decline in anode performance in the SOFC.

## 4. Conclusions

In this study, a multi-physics coupled mesoscale model integrating phase-field modeling and the Lattice Boltzmann Method is developed for the stochastically reconstructed Ni-YSZ anode, enabling prediction of microstructural evolution and performance degradation in direct internal reforming solid oxide fuel cells (SOFCs). The PFM simulates Ni coarsening dynamics, while the LBM assesses methane-steam mass transport characteristics within the anode structure. A comprehensive analysis compares the degrees of Ni coarsening with methane-steam mass transfer performance across varying Ni particle diameters and evolution stages. The main conclusions derived from this study are outlined as follows:

(1) The phase-field simulations demonstrate that Ni particles undergo rapid agglomeration in the early stages (0–2000 steps), forming larger clusters with necking structures, leading to a stable microstructure in later stages (3000–5000 steps). The average Ni particle diameter increases from approximately 0.956 μm to 1.03 μm, with a maximum reaching 1.04 μm, consistent with Ostwald ripening driven by surface energy minimization. This coarsening reduces the fraction of active sites from 3.45% to 2.96%, highlighting a significant loss in electrochemical reaction sites due to decreased surface-to-volume ratio.
(2) The effect of initial Ni particle diameter on coarsening reveals that smaller diameters (e.g., 0.79 μm or 0.70 μm) accelerate agglomeration, resulting in pronounced merging and network formation. In comparison, larger diameters (e.g., 1.20 μm) suppress coarsening, maintaining more dispersed morphologies. Intermediate diameters (e.g., 0.96 μm) exhibit balanced kinetics, with moderate reductions in active site fraction (~14.1% after 10,000 steps). This indicates that the initial particle size governs the coarsening rate through diffusion-limited processes and YSZ constraints, offering insights for tailoring anode microstructures to mitigate degradation.
(3) Ni coarsening impacts mass transport by altering pore connectivity, as evidenced by methane-steam concentration and velocity distributions. In unaggregated states, uniform high-concentration regions support efficient diffusion. However, aggregation introduces localized blockages in necking areas, reducing velocity to near zero and causing heterogeneous flow fields. The local porosity rises from 60% to 62.36% as Ni contraction releases localized space, potentially enhancing overall transport efficiency within macroscopic channels. However, localized blockages in necking regions ultimately compromise anode performance by impeding mass transport.

Based on these findings, an optimal initial Ni particle diameter of approximately 0.96 μm is recommended to mitigate Ni agglomeration and enhance mass transport. This size balances coarsening suppression with sufficient pore connectivity, maintaining higher TPB density and active site fraction while minimizing localized blockages, thereby improving SOFC longevity and efficiency. Future work could extend this model to three-dimensional simulations and incorporate experimental validation to broaden its applicability.

## Figures and Tables

**Table 1:** Parameters for reconstructed Ni–YSZ anode microstructures with varying particle diameter sizes.

Ni-YSZ Region Size	Diameter Ni	Diameter YSZ	Ni Ratio
6 μm × 6 μm × 6 μm	1 μm	0.6 μm	1/6
200 × 200	40	20	1/5
200 × 200	33	20	1/6
200 × 200	29	20	1/7
200 × 200	25	20	1/8

## Data Availability

The original contributions presented in this study are included in the article. Further inquiries can be directed to the corresponding author.
